# Supplementary material for: Proteome-wide signatures of function in highly diverged intrinsically disordered regions
Source: eLife. 2019 Jul 2;8:e46883. doi: 10.7554/eLife.46883 (PMC6634968; doi:10.7554/eLife.46883)
Supplement: Supplementary file 1. [file elife-46883-supp1.docx]

# Supplementary tables

Table S1. Molecular features that have been shown or are hypothesized to be important in IDRs. All motif features are calculated as the fraction of motifs in the IDR normalized to the proteome-wide average. Some motif descriptions taken from Eukaryotic Linear Motif (ELM) resource (Dinkel et al., 2016) – refer to the ELM website for more details: http://elm.eu.org.

|  | ID | Name | Regular expression (regex) | Type | Source | Description | Reference |
| --- | --- | --- | --- | --- | --- | --- | --- |
| 1 | AA_S | S content | S | Amino acid content | NA | Fraction of S residues | (Haynes et al., 2006) |
| 2 | AA_P | P content | P | Amino acid content | NA | Fraction of P residues | (Marsh and Forman-Kay, 2010; Neduva and Russell, 2005; Simon and Hancock, 2009) |
| 3 | AA_T | T content | T | Amino acid content | NA | Fraction of T residues | Reviewed in (Van Der Lee et al., 2014) |
| 4 | AA_A | A content | A | Amino acid content | NA | Fraction of A residues | (Perez et al., 2014) |
| 5 | AA_H | H content | H | Amino acid content | NA | Fraction of H residues | (Marsh and Forman-Kay, 2010) |
| 6 | AA_Q | Q content | Q | Amino acid content | NA | Fraction of Q residues | (Alberti et al., 2009; Halfmann et al., 2011) |
| 7 | AA_N | N content | N | Amino acid content | NA | Fraction of N residues | (Alberti et al., 2009; Halfmann et al., 2011) |
| 8 | AA_G | G content | G | Amino acid content | NA | Fraction of G residues | (Elbaum-Garfinkle et al., 2015) |
| 9 | kappa | Kappa | NA | Charge properties | localCIDER | Measure of separation between positively versus negatively charged residues | (Das and Pappu, 2013; Holehouse et al., 2017) |
| 10 | omega | Omega | NA | Charge properties | localCIDER | Measure of separation between charged residues and prolines versus all other residues | (Holehouse et al., 2017; Martin et al., 2016) |
| 11 | FCR | Fraction of charged residues | NA | Charge properties | localCIDER | FCR: basic fraction + acidic fraction | (Holehouse et al., 2017; Mao et al., 2013) |
| 12 | NCPR | Net charge per residue | NA | Charge properties | localCIDER | NCPR: basic fraction - acidic fraction | (Holehouse et al., 2017; Mao et al., 2013, 2010) |
| 13 | net_charge | net charge | NA | Charge properties | Literature /localCIDER | Net charge (# [RK] - # [DE]) | (Daughdrill et al., 2007; Strickfaden et al., 2007; Zarin et al., 2017) |
| 14 | net_charge_P | net charge with phosphorylation of [ST]P consensus sites | NA | Charge properties | Literature | Net charge as influenced by phosphorylation of consensus sites | (Strickfaden et al., 2007; Zarin et al., 2017) |
| 15 | SCD | Sequence charge decoration | NA | Charge properties | Literature | Measure of separation between positively versus negatively charged residues | (Sawle and Ghosh, 2015) |
| 16 | RK_ratio | R/K ratio | NA | Charge properties | Literature | Ratio of arginine to lysine residues (#R + 1) /(#K + 1) | (Vernon et al., 2018) |
| 17 | ED_ratio | E/D ratio | NA | Charge properties | NA | Ratio of glutamic acid to aspartic acid residues (#E + 1)/(#D + 1) | NA |
| 18 | CLV_Separin_Fungi | Separase cleavage motif | S[IVLMH]E[IVPFMLYAQR]GR. | Motifs | ELM | Separase cleavage site, best known in sister chromatid separation. Also involved in stabilizing the anaphase spindle and centriole disengagement. | (Dinkel et al., 2016) |
| 19 | DEG_APCC_KENBOX_2 | APCC-binding Destruction motif | .KEN. | Motifs | ELM | Motif conserving the exact sequence KEN that binds to the APC/C subunit Cdh1 causing the protein to be targeted for 26S proteasome mediated degradation. | (Dinkel et al., 2016) |
| 20 | DEG_APCC_TPR_1 | APCC_TPR-docking motif | .[ILM]R | Motifs | ELM | This short C-terminal motif is present in co-activators, the Doc1/APC10 subunit and some substrates of the APC/C and mediates direct binding to TPR-containing APC/C core subunits. | (Dinkel et al., 2016) |
| 21 | DOC_CKS1_1 | Cks1 ligand | [MPVLIFWYQ].(T)P.. | Motifs | ELM | Phospho-dependent motif that mediates docking of CDK substrates and regulators to cyclin-CDK-bound Cks1. | (Dinkel et al., 2016) |
| 22 | DOC_MAPK_DCC_7 | MAPK docking motif | [RK].{2,4}[LIVP]P.[LIV].[LIVMF]\|[RK].{2,4}[LIVP].P[LIV].[LIVMF] | Motifs | ELM | A kinase docking motif mediating interaction towards the ERK1/2 and p38 subfamilies of MAP kinases | (Dinkel et al., 2016) |
| 23 | DOC_MAPK_gen_1 | MAPK docking motif | [KR]{0,2}[KR].{0,2}[KR].{2,4}[ILVM].[ILVF] | Motifs | ELM | MAPK interacting molecules (e.g. MAPKKs, substrates, phosphatases) carry docking Motifs that help to regulate specific interaction in the MAPK cascade. The classic Motifs approximates (R/K)xxxx#x# where # is a hydrophobic residue. | (Dinkel et al., 2016) |
| 24 | DOC_MAPK_HePTP_8 | MAPK docking motif | ([LIV][^P][^P][RK]....[LIVMP].[LIV].[LIVMF])\|([LIV][^P][^P][RK][RK]G.{4,7}[LIVMP].[LIV].[LIVMF]) | Motifs | ELM | A kinase docking motif that interacts with the ERK1/2 and p38 subfamilies of MAP kinases. | (Dinkel et al., 2016) |
| 25 | DOC_PP1_RVXF_1 | PP1-docking motif RVXF | ..[RK].{0,1}[VIL][^P][FW]. | Motifs | ELM | Protein phosphatase 1 catalytic subunit (PP1c) interacting Motifs binds targeting proteins that dock to the substrate for dephosphorylation. The motif defined is [RK]{0,1}[VI][^P][FW]. | (Dinkel et al., 2016) |
| 26 | DOC_PP2B_PxIxI_1 | Calcineurin (PP2B)-docking motif PxIxI | .P[^P]I[^P][IV][^P] | Motifs | ELM | Calcineurin substrate docking site, leads to the effective dephosphorylation of serine/threonine phosphorylation sites. | (Dinkel et al., 2016) |
| 27 | LIG_APCC_Cbox_2 | APC/C_Apc2-docking motif | DR[YFH][ILFVM][PA].. | Motifs | ELM | Motifs in APC/C co-activators that mediates binding to the APC/C core, possibly the catalytic Apc2 subunit. This second variant defines the motif in APC/C co-activators from TAXON:4751 and TAXON:554915. | (Dinkel et al., 2016) |
| 28 | LIG_AP_GAE_1 | Gamma-adaptin ear interaction motif | [DE][DES][DEGAS]F[SGAD][DEAP][LVIMFD] | Motifs | ELM | The acidic Phe motif mediates the interaction between a set of accessory proteins and the gamma-ear domain (GAE) of GGAs and AP-1. Proposed roles: in clathrin localization and assembly on TGN/endosome membranes and in traffic between the TGN and endosome. | (Dinkel et al., 2016) |
| 29 | LIG_CaM_IQ_9 | Helical calmodulin binding motif | [ACLIVTM][^P][^P][ILVMFCT]Q[^P][^P][^P][RK][^P]{4,5}[RKQ][^P][^P] | Motifs | ELM | Helical peptide motif responsible for Ca2+-independent binding of the CaM . The motif is manly characterized by a hydrophobic residue at position 1, a highly conserved Gln at position 2, basic charges at positions 6 and 11, and a variable Gly at position 7 | (Dinkel et al., 2016) |
| 30 | LIG_EH_1 | EH ligand | .NPF. | Motifs | ELM/PhyloHMM | NPF motif interacting with EH domains, usually during regulation of endocytotic processes | (Dinkel et al., 2016) |
| 31 | LIG_eIF4E_1 | eIF4E binding motif | Y....L[VILMF] | Motifs | ELM | Motif binding to the dorsal surface of eIF4E. | (Dinkel et al., 2016) |
| 32 | LIG_GLEBS_BUB3_1 | GLEBS motif | [EN][FYLW][NSQ].EE[ILMVF][^P][LIVMFA] | Motifs | ELM | Gle2-binding-sequence motif | (Dinkel et al., 2016) |
| 33 | LIG_LIR_Gen_1 | Atg8 protein family ligands | [EDST].{0,2}[WFY]..[ILV] | Motifs | ELM | Canonical LIR motif that binds to Atg8 protein family members to mediate processes involved in autophagy. | (Dinkel et al., 2016) |
| 34 | LIG_PCNA_PIPBox_1 | PCNA binding PIP box | ((^.{0,3})\|(Q)).[^FHWY][ILM][^P][^FHILVWYP][HFM][FMY].. | Motifs | ELM/PhyloHMM | The PCNA binding PIP box motif is found in proteins involved in DNA replication, repair and cell cycle control. | (Dinkel et al., 2016) |
| 35 | LIG_SUMO_SIM_par_1 | SUMO interaction site | [DEST]{0,5}.[VILPTM][VIL][DESTVILMA][VIL].{0,1}[DEST]{1,10} | Motifs | ELM | Motif for the parallel beta augmentation mode of non-covalent binding to SUMO protein. | (Dinkel et al., 2016) |
| 36 | MOD_CDK_SPxK_1 | CDK Phosphorylation Site | ...([ST])P.[KR] | Motifs | ELM/Condens | Canonical version of the CDK phosphorylation site which shows specificity towards a lysine/arginine residue at the [ST]+3 position. | (Dinkel et al., 2016) |
| 37 | MOD_LATS_1 | LATS kinase phosphorylation motif | H.[KR]..([ST])[^P] | Motifs | ELM | The LATS phosphorylation motif is recognised by the LATS kinases for Ser/Thr phosphorylation. Substrates are often found toward the end of the Hippo signalling pathway. | (Dinkel et al., 2016) |
| 38 | MOD_SUMO_for_1 | Sumoylation site | [VILMAFP](K).E | Motifs | ELM | Motif recognised for modification by SUMO-1 | (Dinkel et al., 2016) |
| 39 | TRG_ER_FFAT_1 | FFAT motif | [DE].{0,4}E[FY][FYK]D[AC].[ESTD] | Motifs | ELM | VAP-A/Scs2 MSP-domain binding FFAT (diphenylalanine [FF] in an Acidic Tract) motif | (Dinkel et al., 2016) |
| 40 | TRG_Golgi_diPhe_1 | ER export signals | Q.{6,6}FF.{6,7} | Motifs | ELM | ER to Golgi anterograde transport signal found at the C-terminus of type I ER-CGN integral membrane cargo receptors (cytoplasmic in this topology), it binds to COPII. | (Dinkel et al., 2016) |
| 41 | TRG_NLS_MonoExtN_4 | NLS classical Nuclear Localization Signals | (([PKR].{0,1}[^DE])\|([PKR]))((K[RK])\|(RK))(([^DE][KR])\|([KR][^DE]))[^DE] | Motifs | ELM | Monopartite variant of the classical basically charged NLS. N-extended version. | (Dinkel et al., 2016) |
| 42 | MOD_CDK_STP | CDK phosphorylation motif | [ST]P | Motifs | Condens | NA | (Holt et al., 2009; A. C. W. Lai et al., 2012) |
| 43 | MOD_MEC1 | Mec1 phosphorylation motif | [ST]Q | Motifs | Condens | NA | (A. C. W. Lai et al., 2012; Schwartz et al., 2002) |
| 44 | MOD_PRK1 | Prk1 phosphorylation motif | [LIVM]….TG | Motifs | Condens | NA | (Huang et al., 2003; A. C. W. Lai et al., 2012) |
| 45 | MOD_IPL1 | Ipl1 phosphorylation motif | [RK].[ST][LIV] | Motifs | Condens | NA | (Cheeseman et al., 2002; A. C. W. Lai et al., 2012) |
| 46 | MOD_PKA | Pka phosphorylation motif | R[RK].S | Motifs | Condens | NA | (Budovskaya et al., 2005; Kemp and Pearson, 1990; A. C. W. Lai et al., 2012; Townsend et al., 1996) |
| 47 | MOD_CKII | Ckii phosphorylation motif | [ST][DE].[DE] | Motifs | Condens | NA | (A. C. W. Lai et al., 2012; Meggio and Pinna, 2003; Niefind et al., 2007) |
| 48 | MOD_IME2 | Ime2 phosphorylation motif | RP.[ST] | Motifs | Condens | NA | (Holt et al., 2007; J. Lai et al., 2012) |
| 49 | DOC_PRO | proline-rich motif | P..P | Motifs | PhyloHMM | NA | (Nguyen Ba et al., 2012) |
| 50 | TRG_ER_HDEL | ER localization motif | HDEL | Motifs | PhyloHMM | NA | (Nguyen Ba et al., 2012) |
| 51 | TRG_MITOCHONDRIA | Mitochondrial localization motif | [MR]L[RK] | Motifs | PhyloHMM | NA | (Nguyen Ba et al., 2012) |
| 52 | MOD_ISOMERASE | Disulfide isomerase motif | C..C | Motifs | PhyloHMM | NA | (Nguyen Ba et al., 2012) |
| 53 | TRG_FG | FG nucleoporin motif | F.FG\|GLFG | Motifs | PhyloHMM | NA | (Frey and Görlich, 2009; Nguyen Ba et al., 2012) |
| 54 | INT_RGG | RGG motif | RGG \| RG | Motifs | Literature | NA | (Chong et al., 2018) |
| 55 | length | Length | NA | Physicochemical properties | Literature | Length in log scale | Reviewed in van der Lee et al. 2014 |
| 56 | acidic | Acidic residue content | [DE] | Physicochemical properties | Literature /localCIDER | NA | (Warren and Shechter, 2017) |
| 57 | basic | Basic residue content | [RK] | Physicochemical properties | Literature /localCIDER | NA | (Fukasawa et al., 2015) |
| 58 | hydrophobicity | Hydrophobicity | NA | Physicochemical properties | Literature /localCIDER | Kyte-Doolittle scale | (Kyte and Doolittle, 1982) |
| 59 | aliphatic | Aliphatic residue content | [ALMIV] | Physicochemical properties | Literature /localCIDER | NA | (Holehouse et al., 2017) |
| 60 | polar_fraction | Polar residue content | [QNSTGCH] | Physicochemical properties | Literature /localCIDER | NA | (Holehouse et al., 2017) |
| 61 | chain_expanding | Chain expanding residue content | [EDRKP] | Physicochemical properties | Literature /localCIDER | NA | (Holehouse et al., 2017) |
| 62 | aromatic | Aromatic residue content | [FYW] | Physicochemical properties | Literature /localCIDER | NA | (Holehouse et al., 2017) |
| 63 | disorder_promoting | Disorder promoting residue content | [TAGRDHQKSEP] | Physicochemical properties | Literature /localCIDER | NA | (Holehouse et al., 2017) |
| 64 | Iso_point | Isoelectric point | NA | Physicochemical properties | Literature /localCIDER | pH where charge of peptide is neutral | (Holehouse et al., 2017; Marsh and Forman-Kay, 2010; Tomasso et al., 2016) |
| 65 | PPII_prop | PPII propensity | NA | Physicochemical properties | Literature /localCIDER | Propensity for proline to form left-handed helices | (Elam et al., 2013; Holehouse et al., 2017) |
| 66 | REP_Q2 | Q repeat | Q{2,} | Repeats and complexity | Literature | Fraction of 2 or more Q in a row | (Chavali et al., 2017) |
| 67 | REP_N2 | N repeat | N{2,} | Repeats and complexity | Literature | Fraction of 2 or more N in a row | (Chavali et al., 2017) |
| 68 | REP_S2 | S repeat | S{2,} | Repeats and complexity | Literature | Fraction of 2 or more S in a row | (Chavali et al., 2017) |
| 69 | REP_G2 | G repeat | G{2,} | Repeats and complexity | Literature | Fraction of 2 or more G in a row | (Chavali et al., 2017). |
| 70 | REP_E2 | E repeat | E{2,} | Repeats and complexity | Literature | Fraction of 2 or more E in a row | (Chavali et al., 2017) |
| 71 | REP_D2 | D repeat | D{2,} | Repeats and complexity | Literature | Fraction of 2 or more D in a row | (Chavali et al., 2017) |
| 72 | REP_K2 | K repeat | K{2,} | Repeats and complexity | Literature | Fraction of 2 or more K in a row | (Matsushima et al., 2009; Simon and Hancock, 2009) |
| 73 | REP_R2 | R repeat | R{2,} | Repeats and complexity | Literature | Fraction of 2 or more R in a row | (Matsushima et al., 2009; Simon and Hancock, 2009) |
| 74 | REP_P2 | P repeat | P{2,} | Repeats and complexity | Literature | Fraction of 2 or more P in a row | (Chavali et al., 2017; Matsushima et al., 2009; Simon and Hancock, 2009) |
| 75 | REP_QN2 | Q/N repeat | [QN]{2,} | Repeats and complexity | Literature | Fraction of 2 or more Q/N in a row | (Alberti et al., 2009; Van Der Lee et al., 2014) |
| 76 | REP_RG2 | R/G repeat | [RG]{2,} | Repeats and complexity | Literature | Fraction of 2 or more R/G in a row; aka "GAR" regions | (Chong et al., 2018; Matsushima et al., 2009) |
| 77 | REP_FG2 | F/G repeat | [FG]{2,} | Repeats and complexity | Literature | Fraction of 2 or more F/G in a row | Reviewed in (Van Der Lee et al., 2014) |
| 78 | REP_SG2 | S/G repeat | [SG]{2,} | Repeats and complexity | Literature | Fraction of 2 or more S/G in a row | (Matsushima et al., 2009; Simon and Hancock, 2009) |
| 79 | REP_SR2 | S/R repeat | [SR]{2,} | Repeats and complexity | Literature | Fraction of 2 or more S/R in a row | Reviewed in (Van Der Lee et al., 2014) |
| 80 | REP_KAP2 | K/A/P repeat | [KAP]{2,} | Repeats and complexity | Literature | Fraction of 2 or more K/A/P in a row | Reviewed in (Van Der Lee et al., 2014) |
| 81 | REP_PTS2 | P/T/S repeat | [PTS]{2,} | Repeats and complexity | Literature | Fraction of 2 or more P/T/S in a row | Reviewed in (Van Der Lee et al., 2014) |
| 82 | wf_complexity | Wootton-Federhen sequence complexity | NA | Repeats and complexity | Literature /localCIDER | Complexity based on SEG algorithm (Wootton and Federhen, 1993), blob length=IDR length, step size = 1 | (Wootton and Federhen, 1993) |

Table S2. Controls for clustering results.

| Cluster ID | Random permutation z-score | Amino acid permutation z-score | Percent of homologous IDRs (top 1% homology in proteome) |
| --- | --- | --- | --- |
| A | 24.94 | 6.13 | 1.47 |
| B | 10.21 | 8.99 | 0 |
| C | 30.77 | 10.74 | 0 |
| D | 38.02 | 22.07 | 1.23 |
| E | 7.87 | 6.54 | 0 |
| F | 15.45 | 12.74 | 0 |
| G | 12.99 | 9.41 | 5.87 |
| H | 29.01 | 14.35 | 0 |
| I | 19.88 | 11.37 | 0 |
| J | 28.05 | 8.62 | 0 |
| K | 7.9 | 9.95 | 0 |
| L | 45.49 | 11.62 | 0.43 |
| M | 47.5 | 15.31 | 2.84 |
| N | 55.28 | 23.98 | 0 |
| O | 46.42 | 7.16 | 0.6 |
| P | 50.78 | 22.18 | 0.26 |
| Q | 230.85 | 50.28 | 8.86 |
| R | 16.51 | 5.97 | 0.94 |
| S | 19.74 | 21.84 | 0 |
| T | 13.77 | 10.43 | 0 |
| U | 16.81 | 8.15 | 0.03 |
| V | 44.33 | 10.41 | 0 |
| W | 187.24 | 39.2 | 0 |

Table S3. Functional annotation based on clustering of evolutionary signatures of fully disordered proteins matches known description of gene function for 5/10 proteins (*). Other proteins where the gene description does not match the gene description exactly, but the Cluster ID is consistent with the gene description are indicated with +.

| ID | Name | Description | % Disorder | Cluster ID |
| --- | --- | --- | --- | --- |
| YNL327W | EGT2 | Glycosylphosphatidylinositol (GPI)-anchored cell wall endoglucanase; required for proper cell separation after cytokinesis; expression is activated by Swi5p and tightly regulated in a cell cycle-dependent manner | 100 | Q: Cell wall organization (*) |
| YNR044W | AGA1 | Anchorage subunit of a-agglutinin of a-cells; highly O-glycosylated protein with N-terminal secretion signal and C-terminal signal for addition of GPI anchor to cell wall, linked to adhesion subunit Aga2p via two disulfide bonds; AGA1 has a paralog, FIG2, that arose from the whole genome duplication | 100 | Q: Cell wall organization (*) |
| YPL163C | SVS1 | Cell wall and vacuolar protein; required for wild-type resistance to vanadate; SVS1 has a paralog, SRL1, that arose from the whole genome duplication | 100 | Q: Cell wall organization (*) |
| YCR089W | FIG2 | Cell wall adhesin, expressed specifically during mating; may be involved in maintenance of cell wall integrity during mating; FIG2 has a paralog, AGA1, that arose from the whole genome duplication | 99 | Q: Cell wall organization (*) |
| YDR077W | SED1 | Major stress-induced structural GPI-cell wall glycoprotein; associates with translating ribosomes, possible role in mitochondrial genome maintenance; ORF contains two distinct variable minisatellites; SED1 has a paralog, SPI1, that arose from the whole genome duplication | 99 | P: Signal transduction (+) |
| YBR108W | AIM3 | Protein that inhibits barbed-end actin filament elongation; interacts with Rvs167p; null mutant is viable and displays elevated frequency of mitochondrial genome loss | 98 | O: Sup35-like |
| YMR219W | ESC1 | Protein involved in telomeric silencing; required for quiescent cell telomere hypercluster localization at nuclear membrane vicinity; interacts with PAD4-domain of Sir4p | 98 | A: Ribosome biogenesis (+) |
| YER167W | BCK2 | Serine/threonine-rich protein involved in PKC1 signaling pathway; protein kinase C (PKC1) signaling pathway controls cell integrity; overproduction suppresses pkc1 mutations | 97 | M: Nucleocytoplasmic transport |
| YDR192C | NUP42 | FG-nucleoporin component of central core of the nuclear pore complex; also part of the nuclear pore complex (NPC) cytoplasmic filaments; contributes directly to nucleocytoplasmic transport and maintenance of the NPC permeability barrier and is involved in gene tethering at the nuclear periphery; interacts with Gle1p; human homolog NUP42 can complement yeast mutant | 97 | M: Nucleocytoplasmic transport (*) |
| YDL223C | HBT1 | Shmoo tip protein, substrate of Hub1p ubiquitin-like protein; mutants are defective for mating projection formation, thereby implicating Hbt1p in polarized cell morphogenesis; HBT1 has a paralog, YNL195C, that arose from the whole genome duplication | 97 | H: Cytoplasmic stress granule |

Table S4. List of strains used in this study.

| Strain | Genotype | Source |
| --- | --- | --- |
| YTZ113 | MATa his3Δ1 leu2Δ0 ura3Δ0 met15Δ0 Cox15-GFP-His3 | Huh et al., courtesy of Brenda Andrews' lab |
| YTZ115 | MATa his3Δ1 leu2Δ0 ura3Δ0 met15Δ0 Mdl2-GFP-His3 | Huh et al., courtesy of Brenda Andrews' lab |
| YBS270 | MATa his3Δ1 leu2Δ0 ura3Δ0 met15Δ0 Cox15-GFP-His3 Cox15 IDR (a.a. 1-45)::0 | This study |
| YBS271 | MATa his3Δ1 leu2Δ0 ura3Δ0 met15Δ0 Cox15-GFP-His3 Cox15 IDR (a.a. 1-45)::Atm1 IDR (a.a. 1-84) | This study |
| YBS272 | MATa his3Δ1 leu2Δ0 ura3Δ0 met15Δ0 Mdl2-GFP-His3 Mdl2 IDR (a.a. 1-99)::0 | This study |
| YBS273 | MATa his3Δ1 leu2Δ0 ura3Δ0 met15Δ0 Mdl2-GFP-His3 Mdl2 IDR (a.a. 1-99)::Atm1 IDR (a.a. 1-84) | This study |
| YBS278 | MATa his3Δ1 leu2Δ0 ura3Δ0 met15Δ0 Cox15-GFP-His3 Cox15 IDR (a.a. 1-45)::Emp47 IDR (a.a. 1-37) | This study |
| YTZ127 | MATa his3Δ1 leu2Δ0 ura3Δ0 met15Δ0 SSK22::HisMX3 SSK2∆0 HO::pFUS1-yemGFP-klURA3 | This study |
| YTZ129 | MATa his3Δ1 leu2Δ0 ura3Δ0 met15Δ0 SSK22::HisMX3 SSK2∆0 Ste50 IDR (a.a. 152-250)::Pex5 IDR (a.a.77-161) HO::pFUS1-yemGFP-klURA3 | This study |
| YTZ130 | MATa his3Δ1 leu2Δ0 ura3Δ0 met15Δ0 SSK22::HisMX3 SSK2∆0 Ste50 IDR (a.a. 152-250)::Rad26 IDR (a.a. 163-269) HO::pFUS1-yemGFP-klURA3 | This study |
| YTZ131 | MATa his3Δ1 leu2Δ0 ura3Δ0 met15Δ0 SSK22::HisMX3 SSK2∆0 Ste50 IDR (a.a. 152-250)::Stp4 IDR (a.a. 144-256) HO::pFUS1-yemGFP-klURA3 | This study |
| YBS292 | MATa his3Δ1 leu2Δ0 ura3Δ0 met15Δ0 Cox15-GFP-His3 Cox15 IDR (a.a. 1-45)::Emp47 IDR (a.a. 1-37) reverted to Cox15 WT IDR | This study |
| YBS293 | MATa his3Δ1 leu2Δ0 ura3Δ0 met15Δ0 Cox15-GFP-His3 Cox15 IDR (a.a. 1-45)::0 reverted to Cox15 WT IDR | This study |

# Supplementary references

Alberti S, Halfmann R, King O, Kapila A, Lindquist S. 2009. A Systematic Survey Identifies Prions and Illuminates Sequence Features of Prionogenic Proteins. *Cell* **137**:146–158. doi:10.1016/j.cell.2009.02.044

Budovskaya Y V., Stephan JS, Deminoff SJ, Herman PK. 2005. An evolutionary proteomics approach identifies substrates of the cAMP-dependent protein kinase. *Proc Natl Acad Sci* **102**:13933–13938. doi:10.1073/pnas.0501046102

Chavali S, Chavali PL, Chalancon G, De Groot NS, Gemayel R, Latysheva NS, Ing-Simmons E, Verstrepen KJ, Balaji S, Babu MM. 2017. Constraints and consequences of the emergence of amino acid repeats in eukaryotic proteins. *Nat Struct Mol Biol* **24**:765–777. doi:10.1038/nsmb.3441

Cheeseman IM, Anderson S, Jwa M, Green EM, Kang J-S, Yates Iii JR, Chan CSM, Drubin DG, Barnes G. 2002. Phospho-Regulation of Kinetochore-Microtubule Attachments by the Aurora Kinase Ipl1p will require the identification of any remaining kineto- chore proteins. Given the central role that kinetochore-microtubule. *Cell* **111**:163–172.

Chong PA, Vernon RM, Forman-Kay JD. 2018. RGG/RG Motif Regions in RNA Binding and Phase Separation. *J Mol Biol* **430**:4650–4665. doi:10.1016/j.jmb.2018.06.014

Das RK, Pappu R V. 2013. Conformations of intrinsically disordered proteins are influenced by linear sequence distributions of oppositely charged residues. *Proc Natl Acad Sci U S A* **110**:13392–7. doi:10.1073/pnas.1304749110

Daughdrill GW, Narayanaswami P, Gilmore SH, Belczyk A, Brown CJ. 2007. Dynamic behavior of an intrinsically unstructured linker domain is conserved in the face of negligible amino acid sequence conservation. *J Mol Evol* **65**:277–288. doi:10.1007/s00239-007-9011-2

Dinkel H, Van Roey K, Michael S, Kumar M, Uyar B, Altenberg B, Milchevskaya V, Schneider M, Kühn H, Behrendt A, Dahl SL, Damerell V, Diebel S, Kalman S, Klein S, Knudsen AC, Mäder C, Merrill S, Staudt A, Thiel V, Welti L, Davey NE, Diella F, Gibson TJ. 2016. ELM 2016—data update and new functionality of the eukaryotic linear motif resource. *Nucleic Acids Res* **44**:D294–D300. doi:10.1093/nar/gkv1291

Elam WA, Schrank TP, Campagnolo AJ, Hilser VJ. 2013. Evolutionary conservation of the polyproline II conformation surrounding intrinsically disordered phosphorylation sites. *Protein Sci* **22**:405–417. doi:10.1002/pro.2217

Elbaum-Garfinkle S, Kim Y, Szczepaniak K, Chen CC-H, Eckmann CR, Myong S, Brangwynne CP. 2015. The disordered P granule protein LAF-1 drives phase separation into droplets with tunable viscosity and dynamics. *Proc Natl Acad Sci* **112**:7189–7194. doi:10.1073/pnas.1504822112

Frey S, Görlich D. 2009. FG/FxFG as well as GLFG repeats form a selective permeability barrier with self-healing properties. *EMBO J* **28**:2554–2567. doi:10.1038/emboj.2009.199

Fukasawa Y, Tsuji J, Fu S-C, Tomii K, Horton P, Imai K. 2015. MitoFates: Improved Prediction of Mitochondrial Targeting Sequences and Their Cleavage Sites. *Mol Cell Proteomics* **14**:1113–1126. doi:10.1074/mcp.M114.043083

Halfmann R, Alberti S, Krishnan R, Lyle N, O’Donnell CW, King OD, Berger B, Pappu R V., Lindquist S. 2011. Opposing Effects of Glutamine and Asparagine Govern Prion Formation by Intrinsically Disordered Proteins. *Mol Cell* **43**:72–84. doi:10.1016/j.molcel.2011.05.013

Haynes C, Oldfield CJ, Ji F, Klitgord N, Cusick ME, Radivojac P, Uversky VN, Vidal M, Iakoucheva LM. 2006. Intrinsic disorder is a common feature of hub proteins from four eukaryotic interactomes. *PLoS Comput Biol* **2**:0890–0901. doi:10.1371/journal.pcbi.0020100

Holehouse AS, Das RK, Ahad JN, Richardson MOG, Pappu R V. 2017. CIDER: Resources to Analyze Sequence-Ensemble Relationships of Intrinsically Disordered Proteins. *Biophys J* **112**:16–21. doi:10.1016/j.bpj.2016.11.3200

Holt LJ, Hutti JE, Cantley LC, Morgan DO. 2007. Evolution of Ime2 phosphorylation sites on Cdk1 substrates provides a mechanism to limit the effects of the phosphatase Cdc14 in meiosis. *Mol Cell* **25**:689–702. doi:10.1016/j.molcel.2007.02.012

Holt LJ, Tuch BB, Villén J, Johnson AD, Gygi SP, Morgan DO. 2009. Global analysis of Cdk1 substrate phosphorylation sites provides insights into evolution. *Science* **325**:1682–6. doi:10.1126/science.1172867

Huang B, Zeng G, Ng AYJ, Cai M. 2003. Identification of novel recognition motifs and regulatory targets for the yeast actin-regulating kinase Prk1p. *Mol Biol Cell* **14**:4871–84. doi:10.1091/mbc.e03-06-0362

Kemp BE, Pearson RB. 1990. Protein kinase recognition sequence motifs. *Trends Biochem Sci* **15**:342–346. doi:10.1016/0968-0004(90)90073-K

Kyte J, Doolittle RF. 1982. A simple method for displaying the hydropathic character of a protein. *J Mol Biol* **157**:105–132. doi:10.1016/0022-2836(82)90515-0

Lai ACW, Nguyen Ba AN, Moses AM. 2012. Predicting kinase substrates using conservation of local motif density. *Bioinformatics* **28**:962–9. doi:10.1093/bioinformatics/bts060

Lai J, Koh CH, Tjota M, Pieuchot L, Raman V, Chandrababu KB, Yang D, Wong L, Jedd G. 2012. Intrinsically disordered proteins aggregate at fungal cell-to-cell channels and regulate intercellular connectivity. *Proc Natl Acad Sci* **109**:15781–15786. doi:10.1073/pnas.1207467109

Mao AH, Crick SL, Vitalis A, Chicoine CL, Pappu R V. 2010. Net charge per residue modulates conformational ensembles of intrinsically disordered proteins. *Proc Natl Acad Sci U S A* **107**:8183–8. doi:10.1073/pnas.0911107107

Mao AH, Lyle N, Pappu R V. 2013. Describing sequence–ensemble relationships for intrinsically disordered proteins. *Biochem J* **449**:307–318. doi:10.1042/BJ20121346

Marsh JA, Forman-Kay JD. 2010. Sequence determinants of compaction in intrinsically disordered proteins. *Biophys J* **98**:2374–2382. doi:10.1016/j.bpj.2010.02.012

Martin EW, Holehouse AS, Grace CR, Hughes A, Pappu R V, Mittag T. 2016. Sequence determinants of the conformational properties of an intrinsically disordered protein prior to and upon multisite phosphorylation. *J Am Chem Soc* jacs.6b10272. doi:10.1021/jacs.6b10272

Matsushima N, Tanaka T, Kretsinger R. 2009. Non-Globular Structures of Tandem Repeats in Proteins. *Protein Pept Lett* **16**:1297–1322. doi:10.2174/092986609789353745

Meggio F, Pinna LA. 2003. One-thousand-and-one substrates of protein kinase CK2? *FASEB J* **17**:349–68. doi:10.1096/fj.02-0473rev

Neduva V, Russell RB. 2005. Linear motifs: Evolutionary interaction switches. *FEBS Lett* **579**:3342–3345. doi:10.1016/j.febslet.2005.04.005

Nguyen Ba AN, Yeh BJ, van Dyk D, Davidson AR, Andrews BJ, Weiss EL, Moses AM. 2012. Proteome-wide discovery of evolutionary conserved sequences in disordered regions. *Sci Signal* **5**:rs1. doi:10.1126/scisignal.2002515

Niefind K, Yde CW, Ermakova I, Issinger OG. 2007. Evolved to Be Active: Sulfate Ions Define Substrate Recognition Sites of CK2α and Emphasise its Exceptional Role within the CMGC Family of Eukaryotic Protein Kinases. *J Mol Biol* **370**:427–438. doi:10.1016/j.jmb.2007.04.068

Perez RB, Tischer A, Auton M, Whitten ST. 2014. Alanine and proline content modulate global sensitivity to discrete perturbations in disordered proteins. *Proteins Struct Funct Bioinforma* **82**:3373–3384. doi:10.1002/prot.24692

Sawle L, Ghosh K. 2015. A theoretical method to compute sequence dependent configurational properties in charged polymers and proteins. *J Chem Phys* **143**. doi:10.1063/1.4929391

Schwartz MF, Duong JK, Sun Z, Morrow JS, Pradhan D, Stern DF. 2002. Rad9 Phosphorylation Sites Couple Rad53 to the Saccharomyces cerevisiae DNA Damage Checkpoint. *Mol Cell* **9**:1055–1065. doi:10.1016/S1097-2765(02)00532-4

Simon M, Hancock JM. 2009. Tandem and cryptic amino acid repeats accumulate in disordered regions of proteins. *Genome Biol* **10**:1–16. doi:10.1186/gb-2009-10-6-r59

Strickfaden SC, Winters MJ, Ben-Ari G, Lamson RE, Tyers M, Pryciak PM. 2007. A mechanism for cell-cycle regulation of MAP kinase signaling in a yeast differentiation pathway. *Cell* **128**:519–31. doi:10.1016/j.cell.2006.12.032

Tomasso ME, Tarver MJ, Devarajan D, Whitten ST. 2016. Hydrodynamic Radii of Intrinsically Disordered Proteins Determined from Experimental Polyproline II Propensities. *PLoS Comput Biol* **12**:1–22. doi:10.1371/journal.pcbi.1004686

Townsend RR, Lipniunas PH, Tulk BM, Verkman AS. 1996. Identification of protein kinase a phosphorylation sites on NBD1 and R domains of CFTR using electrospray mass spectrometry with selective phosphate ion monitoring. *Protein Sci* **5**:1865–1873. doi:10.1002/pro.5560050912

Van Der Lee R, Buljan M, Lang B, Weatheritt RJ, Daughdrill GW, Dunker AK, Fuxreiter M, Gough J, Gsponer J, Jones DT, Kim PM, Kriwacki RW, Oldfield CJ, Pappu R V., Tompa P, Uversky VN, Wright PE, Babu MM. 2014. Classification of intrinsically disordered regions and proteins. *Chem Rev* **114**:6589–6631. doi:10.1021/cr400525m

Vernon RM, Chong PA, Tsang B, Kim TH, Bah A, Farber P, Lin H, Forman-Kay JD. 2018. Pi-Pi contacts are an overlooked protein feature relevant to phase separation. *Elife* **7**:1–48. doi:10.7554/eLife.31486

Warren C, Shechter D. 2017. Fly Fishing for Histones: Catch and Release by Histone Chaperone Intrinsically Disordered Regions and Acidic Stretches. *J Mol Biol* **429**:2401–2426. doi:10.1016/j.jmb.2017.06.005

Wootton JC, Federhen S. 1993. Statistics of local complexity in amino acid sequences and sequence databases. *Comput Chem* **17**:149–163. doi:10.1016/0097-8485(93)85006-X

Zarin T, Tsai CN, Nguyen Ba AN, Moses AM. 2017. Selection maintains signaling function of a highly diverged intrinsically disordered region. *Proc Natl Acad Sci* **114**:E1450–E1459. doi:10.1073/pnas.1614787114
